# Palmitate Activates Autophagy in INS-1E β-Cells and in Isolated Rat and Human Pancreatic Islets

**DOI:** 10.1371/journal.pone.0036188

**Published:** 2012-05-01

**Authors:** Luisa Martino, Matilde Masini, Michela Novelli, Pascale Beffy, Marco Bugliani, Lorella Marselli, Pellegrino Masiello, Piero Marchetti, Vincenzo De Tata

**Affiliations:** 1 Department of Experimental Pathology, University of Pisa, Pisa, Italy; 2 Institute of Clinical Physiology, CNR, University of Pisa, Pisa, Italy; 3 Department of Endocrinology and Metabolism, University of Pisa, Pisa, Italy; Universita Magna-Graecia di Catanzaro, Italy

## Abstract

We have investigated the *in vitro* effects of increased levels of glucose and free fatty acids on autophagy activation in pancreatic beta cells. INS-1E cells and isolated rat and human pancreatic islets were incubated for various times (from 2 to 24 h) at different concentrations of glucose and/or palmitic acid. Then, cell survival was evaluated and autophagy activation was explored by using various biochemical and morphological techniques. In INS-1E cells as well as in rat and human islets, 0.5 and 1.0 mM palmitate markedly increased autophagic vacuole formation, whereas high glucose was ineffective alone and caused little additional change when combined with palmitate. Furthermore, LC3-II immunofluorescence co-localized with that of cathepsin D, a lysosomal marker, showing that the autophagic flux was not hampered in PA-treated cells. These effects were maintained up to 18–24 h incubation and were associated with a significant decline of cell survival correlated with both palmitate concentration and incubation time. Ultrastructural analysis showed that autophagy activation, as evidenced by the occurrence of many autophagic vacuoles in the cytoplasm of beta cells, was associated with a diffuse and remarkable swelling of the endoplasmic reticulum. Our results indicate that among the metabolic alterations typically associated with type 2 diabetes, high free fatty acids levels could play a role in the activation of autophagy in beta cells, through a mechanism that might involve the induction of endoplasmic reticulum stress.

## Introduction

Macroautophagy (hereafter referred to as autophagy) is a physiologically conserved protein degradation system that involves the degradation of cellular components through the lysosomal machinery. Autophagy is a tightly regulated process that is activated in cell growth, development and homeostasis, as it contributes to preserve the balance between synthesis, degradation, and recycling of cellular components [1], [2]. A major role of autophagy is to derive nutrients from endogenous sources to use them for survival purposes under conditions such as starvation or deprivation of growth factors. Indeed, autophagy is rapidly induced under nutritional deprivation in yeast [3] as well as in newborn mice [4], thus appearing as a basic survival strategy in all eukaryotes. In these conditions, autophagy leads to bulk degradation of cytoplasmic components (protein, organelles), whose building blocks are used for energy supply and synthesis of essential components for survival [5]. In addition, autophagy also plays a crucial role in cellular housekeeping because it is able to remove exhausted, redundant or unwanted components. Actually, a low level of constitutive autophagy appears suitable for maintaining the “quality” of proteins and organelles [6]. Hence, autophagy can be generally considered as a cellular protective mechanism against various types of injuries or continuous wear and tear. In this way, autophagy can act as an anti-ageing mechanism [7] support cell remodeling during development [8] and contribute to cellular defense against pathogens [9].

Nevertheless, activation of autophagy might also lead to a form of non-apoptotic cell death which is called “type 2 programmed cell death” or autophagic cell death [10]. Autophagic cell death still remains mainly a morphological definition (i.e. cell death associated with abundant autophagosomes/autolysosomes), as no conclusive evidence is available that a specific mechanism of autophagic death actually occurs [10], [11]. In any case, it appears conceivable that autophagy could possibly promote cell death through altered degradation of cellular constituents, depending on the cellular and environmental context [12].

As a matter of fact, in addition to the physiological role of autophagy, dysregulation of this process has been suggested to play important pathogenetic roles in a variety of diseases processes [13], particularly in conditions of increased cellular stress, likely as the result of the accumulation of damaged molecules and organelles.

In type 2 diabetes mellitus, several evidences indicates that a progressive decrease of β-cell function and β-cell mass is a common feature of the disease [14], [15]. Beta cells, because of their sustained and continuous secretory activity, are chronically exposed to various kinds of stress, originating from misfolded proteins, ER hyperactivity, and damaged mitochondria [16]–[18]. As autophagy could exert a protective effect against ER stress [19], and it is also implicated in the maintenance of mitochondrial function by facilitating mitochondrial turnover (mitophagy) [20], it has been proposed that autophagy plays a crucial role in the maintenance of normal β-cell function and survival and that its dysregulation might contribute to β-cell failure in type 2 diabetes [21]–[23]. Recently, we have reported that human type 2 diabetes pancreatic beta cells may show massive overload of autophagic vacuoles and autophagosomes associated with cell damage, suggesting that altered autophagy might contribute to the loss of beta-cell mass [24].

On the basis of these considerations, in the present study we have explored the ability of elevated glucose and/or free fatty acids concentrations to activate autophagy in both INS-1E β-cell line and in isolated rat and human pancreatic islets.

## Methods

### Ethics statement

Human islets were isolated from pancreata of nondiabetic multiorgan donors with the approval of the Ethics Committee of the University of Pisa. Human pancreata were collected from brain-dead organ donors after informed consent was obtained in writing form from family members. With reference to the isolation of rat islets, the experimental protocol followed the principles of Laboratory Animal Care (US NH publication no. 83–85, revised 1985) and was approved by the Ethical Committee of the University of Pisa.

### Cell culture

INS-1E cells were kindly provided by Prof. C.B. Wollheim of the University of Geneva, Switzerland [25]. INS-1E cells were cultured in a humidified atmosphere containing 5% CO_2_ in complete medium composed of RPMI 1640 supplemented with 10% heat-inactivated fetal calf serum, 1 mM sodium pyruvate, 50 µM 2-mercaptoethanol, 2 mM glutamine, 10 mM HEPES, 100 U/ml penicillin, and 100 µg/ml streptomycin. The maintenance culture was passaged once a week by gentle trypsinisation, and cells were seeded at a density of 4×10^4^ cells/cm^2^, i.e. 3×10^6^ cells, in 75-cm^2^ Falcon bottles with 10 ml complete medium.

### Rat islet isolation

Sprague-Dawley male rats of 200–250 g b.w. (Harlan, Italy) were anesthetized with an intraperitoneal injection of 50 mg/kg b.w. pentobarbital sodium. Pancreatic islets were isolated by the collagenase method using the procedure of pancreatic duct cannulation and density gradient purification described elsewhere [26]. Isolated islets, washed several times with buffer, were maintained in KRB/Hepes buffer containing 5.6 mM glucose until experiments were performed.

### Human islet isolation and culture

Human islets were isolated by enzymatic digestion and density gradient purification from pancreatic samples of multiorgan donors, as detailed elsewhere [27], [28]. Isolated islets were then cultured in M199 medium at 5.5 mmol/l glucose until experiments were performed.

### Palmitic acid solutions

Palmitic acid solutions were prepared according to Karasov et al. [29]. Briefly, PA was dissolved at 70°C in 0.1 M NaOH to obtain a 100 mM stock solution. A 5% (w/v) solution of FFA-free bovine serum albumin (BSA) was prepared in serum-free RPMI medium. Then, a 5 mM PA/BSA mixture was prepared by suitable combination of the two above mentioned solutions. Finally, the mixture was conveniently further diluted in RPMI to obtain the required final concentrations.

### Cytotoxicity

INS-1E cells were seeded in 96-well plates at a density of 4×10^4^ cells/cm^2^. After 48 hours, cells were incubated with fresh medium containing PA (0, 0.1, 0.5 and 1.0 mM), glucose (5, 11, 16.7 and 25 mM) or palmitate+glucose for different times (from 2 to 24 h). Cell survival at the end of the incubation was evaluated on the basis of the cleavage of the tetrazolium salt WST-1 by mitochondrial dehydrogenases, by using a commercially available kit (Cell Proliferation Reagent WST-1. Roche Diagnostics, Germany). Absorbance at 440 nm was measured in a scanning multiwell spectrophotometer Victor^3^ 1420 (Perkin Elmer).

### Electron microscopy evaluation

Electron microscopy studies were performed on INS-1E cells and isolated islets as previously described [27] after 6 and 24 h incubation with 0.5 mM PA and/or 25 mM glucose. In particular, autophagic vacuoles were identified from the presence of organelles and/or cytoplasmic portions surrounded by close double membranes; autophagosomes were identified from the presence of single membrane vacuoles containing organelles with signs of degradation [30], [31]. Morphometric analyses were performed by stereological techniques [32]. In particular volume density of autophagic vacuoles (AV) and rough endoplasmic reticulum (RER) was estimated. Twenty microphotographs of each condition were taken, at an original magnification of ×3000. Negatives were printed and enlarged to a final magnification of ×10000. Volume density values derived from the evaluation of 20–30 different cells for each cell category (in most micrographies there were more than one cell). The cytoplasm was used as reference area. A graticule (11×11) composed of 169 points was placed on the micrographs and the number of points intersecting the autophagic vacuoles (AV) was counted. Volume density of AV and RER was calculated according to the formula : VD = Pi/Pt, where Pi is the number of points within the subcellular component and Pt is the total number of points, and expressed in ml/100 ml of tissue (ml%) [32].

In an additional set of experiments, incubation of human islets with 0.5 mM PA was prolonged up to 48 h, either with or without the addition of 10 ng/ml rapamycin, a potentiator of autophagy. At the ultrastructural level, beta cells of isolated human islets were considered dead on the basis of any of the following criteria: loss of plasma membrane integrity, fragmentation into discrete bodies, engulfment of cell corpse or fragments by adjacent cells. The presence of marked chromatin condensation and/or blebs was considered to be a sign of apoptosis [24].

### Mono-dansyl-cadaverine (MDC) staining

INS-1E cells were seeded in 8-well chamber slides (Lab-Tek II Chamber Slide System, Nalge Nunc Intl. Corp., Naperville, IL, USA) at a density of 5×10^5^/ml. After 48 h, cells were incubated with different concentrations of palmitate (0, 0.1, 0.5 and 1.0 mM), glucose (5, 11, 16.7 and 25 mM) and palmitate+glucose. At the end of the incubation, cells were washed twice with PBS and resuspended in PBS containing 500 µM mono-dansyl-cadaverine (MDC) for 10′ at 37°C. Cells were then washed three times with PBS and immediately analyzed under a fluorescent microscopy (355 nm excitation and 460 nm emission wavelenght).

Ten-20 rat or human isolated pancreatic islets suspended in 500 µl buffer were centrifuged at 1200 rpm for 3 min in a Shandon Cytospin 3 Centrifuge. This is a cell preparation system which uses centrifugal force to deposit cells (or islets in this case) upon a microscopic slide. Islets were fixed on the slides with gluteraldeyde, incubated for 15 min with 50 µM monodansyl cadaverine and immediately analyzed under a fluorescent microscopy (355 nm excitation and 460 nm emission wavelenght).

### LC-3 immunochemistry

INS-1E cells were seeded in 8-well chamber slides (Lab-Tek II Chamber Slide System, Nalge Nunc Intl. Corp., Naperville, IL, USA) at a density of 5×10^5^/ml. After 48 h, cells were incubated with different concentrations of palmitate (0, 0.1, 0.5 and 1.0 mM), glucose (5, 11, 16.7 and 25 mM) and palmitate+glucose. At the end of the incubation, cells were washed twice with PBS on ice and then fixed in 2.5% glutaraldehyde in 0.1 M phosphate buffer for 20 min. Cells were then blocked at room temperature with 8% skimmed milk, 1% triton X-100 in PBS for 30 min and washed twice with PBS. Cells were exposed to anti-LC-3 (Sigma-Aldrich L8918) or anti-Catethepsin D (Santa Cruz Biotechnologies Inc., SC-6486) primary antibodies diluted 1∶500 in 0.8% skimmed milk, 0.1% Triton X-100 in PBS for 1 h. Cells were then washed three times for 5 min in PBS and exposed to a FITC conjugate-goat anti-rabbit igG secondary antibody (Sigma-Aldrich F0382), diluted 1∶300 in PBS for 30 min. Cells were finally washed twice in PBS for 5 min and immediately analyzed under a fluorescent microscopy (355 nm excitation and 460 nm emission wavelenght).

### LC-3 western blot analysis

INS-1E cells were seeded in 6-well plates at a density of 5×10^5^/well. After 48 h, cells were exposed to various concentrations of palmitic acid (0, 0.1, 0.5 and 1.0 mM) for different time periods (2, 6, 12 and 18 h). At the end of the incubation, cells were collected by scraping and centrifuged (2,000 g for 5 min) at 4°C. Cell lysates were prepared by incubation on ice for 20 min in 150 µl lysis buffer containing 50 mM Tris-HCl, pH 7.4, 2 mM sodium orthovanadate, 1 µg/ml leupeptin, 1 µg/ml aprotinin, 1 µg/ml pepstatin, and 1 mM PMSF. After centrifugation (10,000 g for 10 min at 4°C), a 10 µl aliquot was used for protein analysis by the Bradford assay (Biorad). Samples containing the same amount of proteins (15 µg) were loaded onto 12% polyacrylamide gels and transferred to a nitrocellulose membrane (Amersham Biosciences, Piscataway, NJ, USA). As a control for loading, α-tubulin was employed in all samples. The blots were blocked with 5% skimmed milk (Amersham) in TTBS (25 mM Tris-HCl, pH 7.4, 150 mM NaCl, 0.2% Tween 20) for 2 h. Primary antibodies (anti-LC-3 polyclonal antibody, Sigma-Aldrich L8918, 1∶1500; anti-tubulin monoclonal antibody, Sigma-Aldrich T5168, 1∶8000) were added for an overnight incubation at 4°C Then the filter was washed and incubated for 1 h with secondary antibodies (HRP-linked anti-rabbit IgG, Sigma Aldrich, A0545, for LC-3 and HRP-linked anti-mouse IgG, Santa Cruz, SC2004, for α-tubulin). Immunoreactive proteins were detected using a chemiluminescence method (Millipore) and X-OMAT film (Kodak).

### Statistical analysis

All results are expressed as mean ± SEM. Statistical significance was evaluated by using analysis of variance (ANOVA) or Student's *t*-test, when appropriate.

## Results

### Effects of glucose on autophagy activation in INS-1E cells

**Figure 1:**
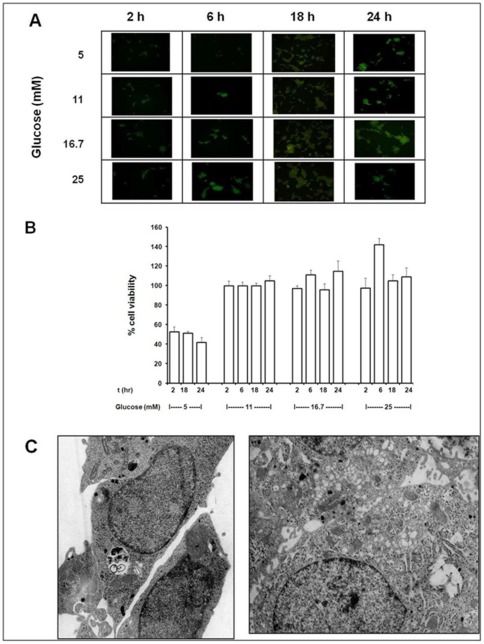
Effects of various incubation times (2–24 h) with different glucose concentrations (5, 11, 16.7 and 25 mM) in INS-1E cells. **A**) Mono-dansyl-cadaverine (MDC) fluorescence, as indicator of autophagosome formation; **B**) cell viability; **C**) ultrastructure after 24 h incubation with 25 mM glucose (magnification: 10.000×). **D**) ultrastructure after 48 h incubation with 30 mM glucose (magnification: 10.000×).

Because glucose is the key physiological regulator of insulin secretion and hyperglycaemia is the metabolic hallmark of diabetes mellitus, we first investigated the effects of increasing glucose concentrations (from 5 to 25 mM) on INS-1E cells after different incubation times (from 2 to 24 h), in terms of both cell viability and autophagy activation. As shown in Fig. 1B, glucose did not reduce the viability of INS-1E cells, with the only exception of the lower concentration (5 mM), in agreement with the well-known dependence of these cells on suitable concentrations of glucose for survival [33]. At the same time, we explored glucose-induced autophagy activation by using monodansyl cadaverine (MDC), an autofluorescent compound used for the *in vivo* labeling of autophagic vacuoles, whose formation is indicated by the appearance of dot-like structures in cytoplasmic and perinuclear regions of MDC-incubated cells. MDC fluorescence, although not completely specific, probably represents the simplest way to detect autophagosome formation, thus allowing a rapid, preliminary screening of autophagy activation in several different experimental conditions, to be subsequently confirmed with other, more sophisticated, methodologies. Our results clearly indicate that no glucose concentration was able to elicit fluorescence-positive dots in the cytoplasm of INS-1E cells (Fig. 1A). These results were confirmed by ultrastructural analysis of these cells, in which no sign of autophagy activation was detectable after incubation with increasing glucose concentrations (e.g., see Fig. 1C, after 24 h at 25 mM glucose). In order to further support these observations, in a separate experiment we explored also the effect of a longer incubation (48 h) with a higher glucose concentration (30 mM). We found actually a significant reduction of cell survival (Control: 100±1.33%; 30 mM glucose: 76.9±3.60%; p<0.05), but even in this drastic experimental condition no signs of autophagy activation were observed, in terms of both MDC fluorescence (data not shown) and electron microscopy analysis (Fig. 1D). In particular, after 48 h exposition to 30 mM glucose, in the cytoplasm of INS-1E cells the insulin granules are almost completely absent, whereas several typical glycogen granules can be detected, either scattered or arranged in quite large clusters. It is important to note that both single and clustered glycogen granules are never delimited by single or double-layer membranes, thus indicating the absence of autophagic degradation. No autophagic vacuoles and only infrequent dense bodies were observed. In agreement with the cell survival data, some cells appear to be frankly apoptotic.

### Effects of palmitic acid on autophagy activation in INS-1E cells

**Figure 2:**
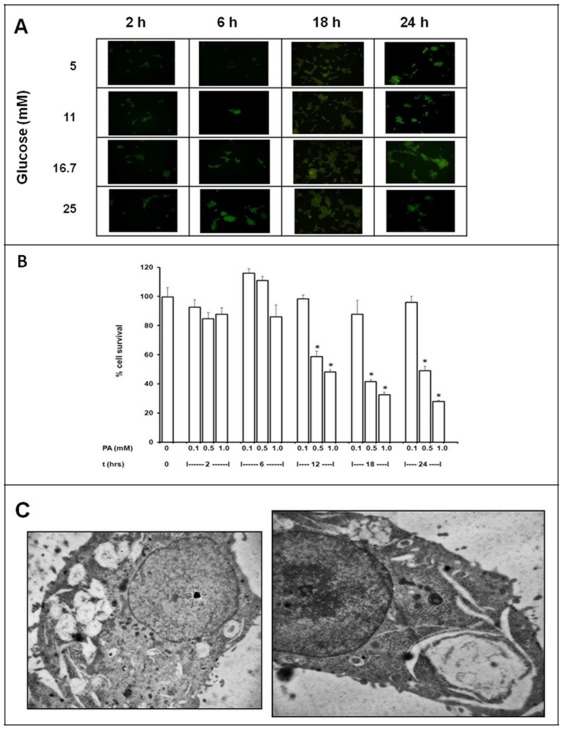
Effects of various incubation times (2–24 h) different palmitic acid (PA) concentrations (0.1, 0.5 and 1.0 mM) in INS-1E cells. **A**) MDC fluorescence, as indicator of autophagosome formation; **B**) cell viability; **C**) ultrastructure (0.5 mM PA for 24 h) (magnification: 10.000×).

Another potential cause of β-cell dysfunction is lipotoxicity, i.e. the deleterious effect on β cells of excessive fatty acids and their metabolic products that occurs in individuals with insulin resistance, glucose intolerance and type 2 diabetes [34]. In particular, the saturated fatty acid palmitic acid (PA) was reported to be cytotoxic to β cells [35]. Thus, we investigated the effects of three PA concentrations (0.1, 0.5 and 1.0 mM) on INS-1E cells after different incubation times (from 2 to 24 h) again evaluating cell viability and autophagy activation. Differently from glucose, PA significantly reduced INS-1E cell viability after 12 h of incubation and thereafter in a dose-dependent fashion (Fig. 2B). Interestingly, the proportion of INS-1E cells containing MDC-stained dots was significantly increased, starting at 6 h after 0.5 and 1 mM PA treatment, as compared to cells unexposed to PA (Fig. 2A). The proportion of MDC-positive cells remained elevated even after 12 and 18 h of PA exposure, slightly declining after 24 h (Fig. 2A). The activation of autophagy by PA in INS-1E was confirmed by electron microscopy. After 24 h of incubation with 0.5 mM PA, in INS-1E cells numerous and large lipid droplets were detectable in the cytoplasm, concomitantly with several autophagic vacuoles and dense bodies. In these cells, enlarged and prevalently perinuclear cysternae were often observed, indicating a remarkable ER stress (Fig. 2C).

**Figure 3:**
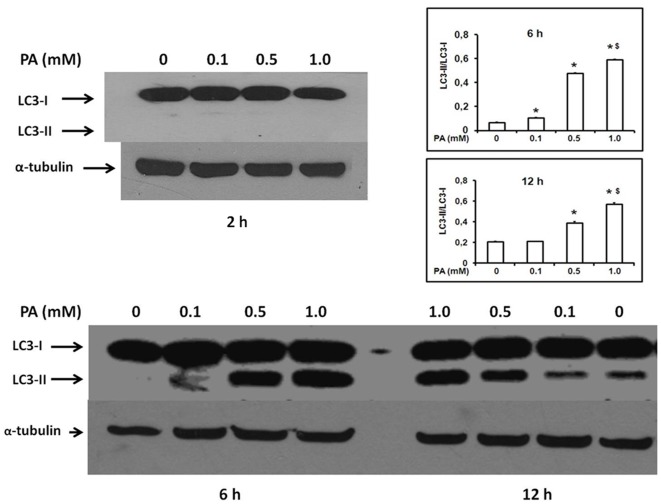
Immunoblotting of LC-3 protein in INS-1E cells after 2, 6 and 12 h treatment with various concentrations of palmitate (PA). α-tubulin was used as a control for loading. Methodological details are described in Materials and Methods. The figure is representative of gels obtained from three separate experiments. From these gels, band intensities were evaluated by densitometric analysis and expressed as the mean ± SEM of the ratio between LC3-II and LC3-I bands (lower graphs). * p<0.01 vs. untreated control; § p<0.01 vs. PA 0.5 mM.

A further confirmation of the ability of PA to induce autophagy activation in INS-1E cells was obtained by immunoblotting showing the proteolytic conversion of the 18-kDa precursor LC3-I into the 16-kDa LC3-II isoform, as expected for true autophagy [36], already 6 h after 0.5 and 1.0 mM PA treatment (Fig. 3). At 12 h, LC3-II band was still clearly increased (in a dose-dependent manner) in PA-treated INS-1E cells with respect to untreated cells. At 18 h a slight increase of LC3-II band was still detectable only in cells exposed to 1 mM PA as compared to controls (not shown).

**Figure 4:**
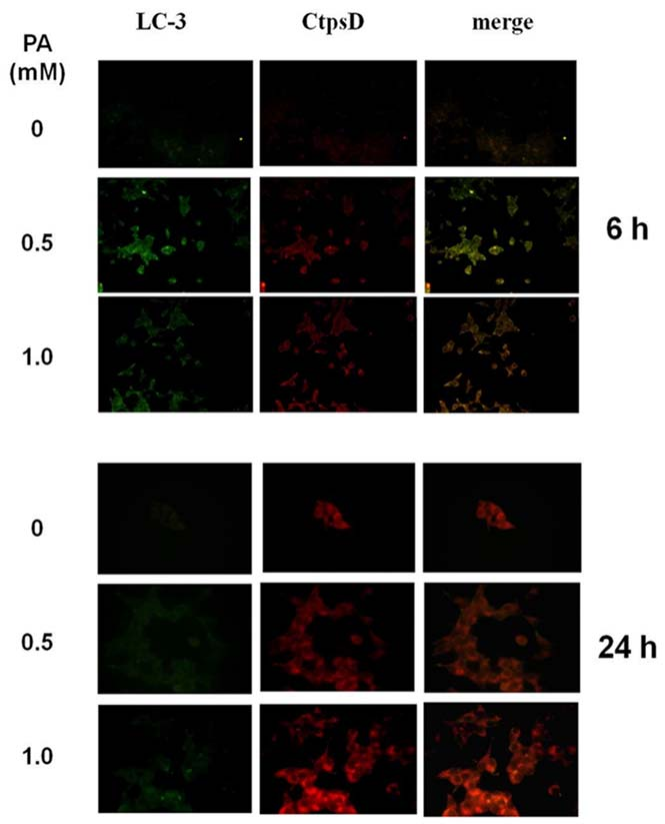
Cellular localization of LC-3 and cathepsin D immunofluorescence in INS-1E cells after 6 and 24 h treatment with 0.5 and 1.0 mM PA.

However, autophagic vacuoles formation, as indicated by MDC fluorescence and LC3-II activation, might not be necessarily followed by their fusion with lysosomes to form autophagolysosomes, in the case authophagic flux is blocked. In order to verify whether autophagosome accumulation resulted from impaired clearance due to defective fusion with lysosomes or from true autophagic flux, we checked for autophagolysosomes formation by double labeling the PA-treated INS-1E cells with LC3, as a marker of autophagosomes, and cathepsin D as a marker of lysosomes. Our results showed the cathepsin D immunofluorescence co-localized with LC3-II immunofluorescence in INS-1E cells 6 h after PA treatment (Fig. 4, upper panel), thus ruling out the possibility that PA hampered autophagosome-lysosome formation. This result does not rule out the possibility of a subsequent impairment of lysosomal degradation. Indeed, it has been recently shown that FFA may reduce lysosomal pH thus impairing autophagy flux [37]. However, 24 h after PA treatment, LC3-II immunofluorescence in INS-1E cells was significantly decreased, whereas cathepsin D immunofluorescence was still clearly evident (Fig. 4, lower panel), most probably indicating that LC3-II itself was degraded as a consequence of the fusion of autophagosomes with lysosomes [38].

When INS-1E cells were exposed to different PA concentrations together with increasing glucose concentrations (up to 25 mM), no additive effects of PA and glucose were observed in terms of both MDC fluorescence, cell survival and ultrastructural changes (not shown).

### Effects of palmitic acid on autophagy activation in isolated pancreatic islets

Although insulin-secreting INS-1E cells, derived from an X-rays-induced rat insulinoma, are generally deemed to be a reliable beta-cell surrogate [39], we wanted to corroborate our results in a more physiological model by using isolated pancreatic islets, which can be considered as a real endocrine micro-organ [39].

**Figure 5:**
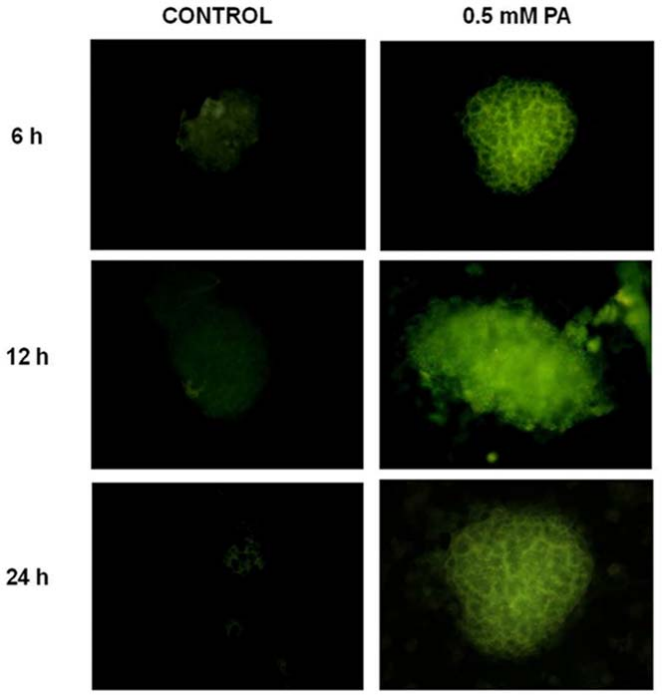
Mono-dansyl-cadaverine (MDC) fluorescence in isolated rat islets exposed for 6, 12 and 24 h to 0.5 mM PA. Isolated islets, previously loaded with the fluorescent probe, were deposited on microscope slides by centrifugation with a cytocentrifuge, as further detailed in Materials and Methods.

To allow the microscopic investigation of pancreatic islets previously loaded with a fluorescent probe such as MDC, we adapted to the islets a method of cellular preparation which employs the centrifugal force of a cytocentrifuge to deposit cells on microscope slides. By using this method, we confirmed that after 6, 12 and 24 h incubation with 0.5 mM PA, the formation of autophagosomes is clearly enhanced in isolated rat islets as evaluated by MDC fluorescence (Fig. 5).

**Figure 6:**
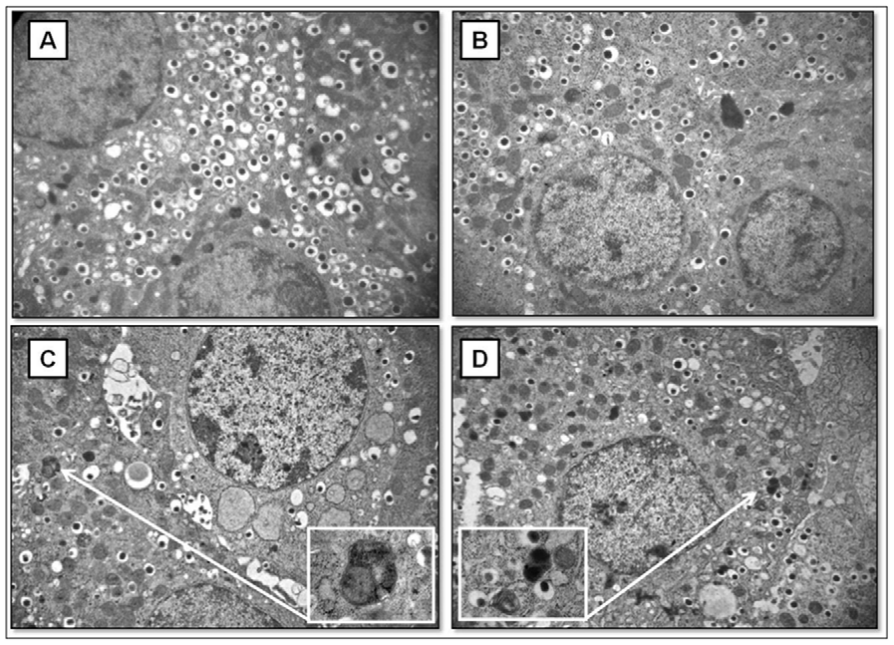
Electron microscopy of isolated rat islets after 6 h incubation with glucose and/or palmitate. **A**) control; **B** 25 mM glucose; **C**) 0.5 mM PA; **D**) 25 mM glucose+0.5 mM pA (magnification: 10.000×; insert: 38.000×).

**Figure 7:**
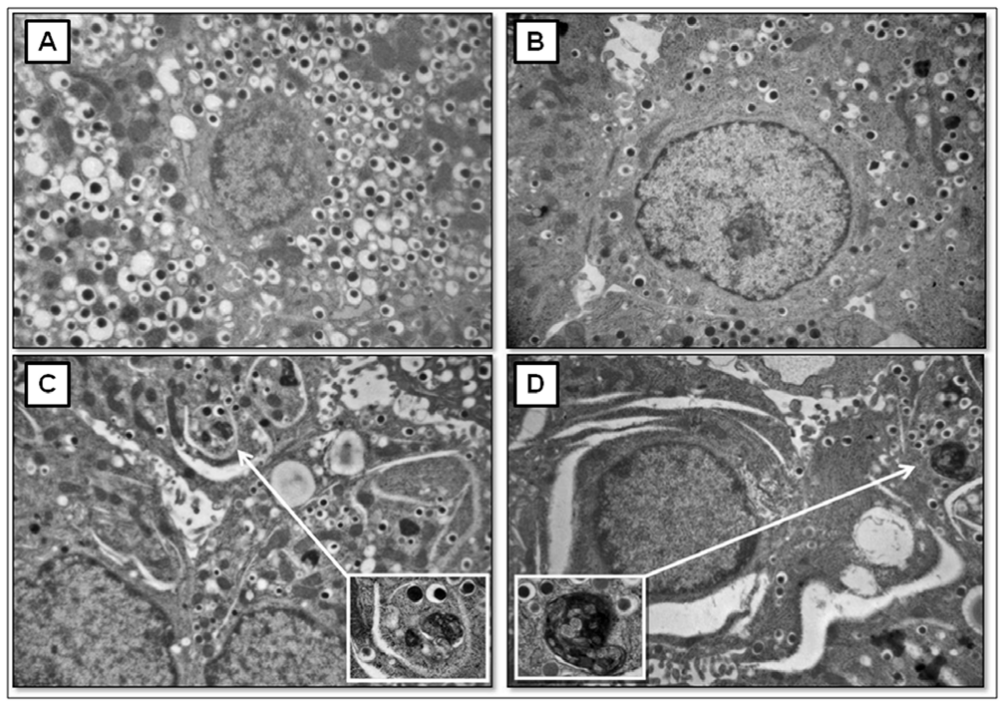
Electron microscopy of isolated rat islets after 24 h incubation with glucose and/or palmitate. **A**) control; **B** 25 mM glucose; **C**) 0.5 mM PA; **D**) 25 mM glucose+0.5 mM pA (magnification: 10.000×; insert: 38.000×).

Ultrastructural analysis of isolated rat islets revealed that after 6 h exposition to 25 mM glucose caused a considerable reduction of secretory granules occurred in beta cells, but no sign of autophagy activation was detected (Fig. 6B). No further alterations were detected in beta cells of isolated rat islets after 24 h exposition to 25 mM glucose (Fig. 7B). Conversely, after 6 h of exposition to 0.5 mM PA, beta cells of isolated rat islets appeared normally granulated, but a diffusely dilated RER and some quite large autophagic vacuoles could be observed in the cytoplasm (Fig. 6C). After 24 h incubation with PA, a remarkable and diffuse RER dilation developed through the whole cytoplasm of beta cells. Furthermore, several autophagic vacuoles containing beta granules and membrane residues of likely mitochondrial derivation were frequently detected between the dilated ER cysternae (Fig. 7C). When the islets were exposed to 25 mM glucose plus 0.5 mM PA for 6 h, the presence of autophagic vacuoles and dilated RER in beta cells was associated with a reduction of the number of secretory granules (Fig. 6D). The combined exposition to glucose and PA for 24 h resulted in increased severity of the above mentioned alterations (Fig. 7D).

As regards to isolated human islets, fluorescence-based experiments unfortunately did not give reliable results because a strong fluorescent signal was observed also in control islets, probably due to interference with the intrinsic autofluorescence of human beta cells [40].

Thereupon, we performed an ultrastructural analysis of human isolated islets after 6 and 24 h exposition to 0.5 mM PA and/or 25 mM glucose.

**Figure 8:**
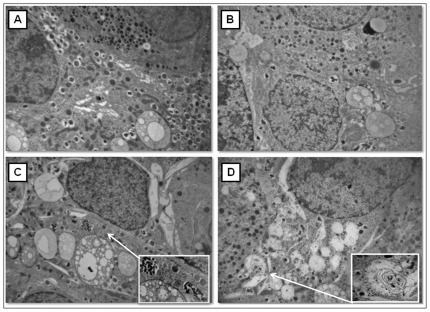
Electron microscopy of isolated human islets after 6 h incubation with glucose and/or palmitate. **A**) control; **B** 25 mM glucose; **C**) 0.5 mM PA; **D**) 25 mM glucose+0.5 mM pA (magnification: 10.000×; insert: 38.000×).

**Figure 9:**
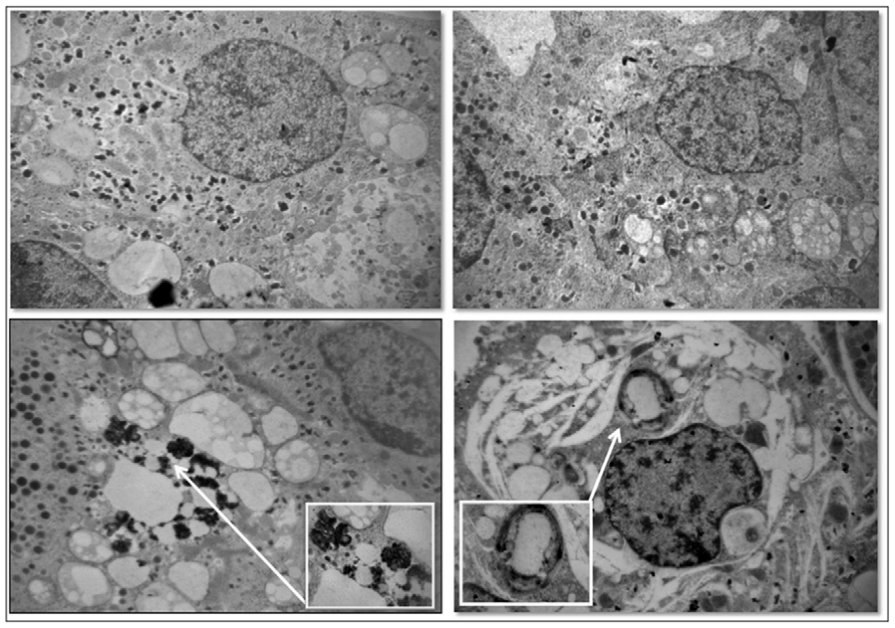
Electron microscopy of isolated human islets after 24 h incubation with glucose and/or palmitate. **A**) control; **B** 25 mM glucose; **C**) 0.5 mM PA; **D**) 25 mM glucose+0.5 mM pA (magnification: 10.000×; insert: 38.000×).

After 6 h in the presence of 25 mM glucose, beta cells in isolated human islets showed no relevant alterations (Fig. 8B): only the secretory granules appeared to be slightly diminished in number. No necrotic or apoptotic cells were detected. A substantially similar picture was observed also when exposure to high glucose was prolonged to 24 h (Fig. 9 B). Conversely, after 6 h in the presence of 0.5 mM PA, beta cells in human islets appeared degranulated and showed a markedly dilated RER that extended through the entire cytoplasm. Mitochondria were frequently swollen. Several autophagic vacuoles containing lipid-like droplets and sometimes beta granule dense cores could be observed. However, no necrotic or apoptotic cells were detected (Fig. 8C). After 24 h in the presence of PA, the ultrastructural alterations were more severe than after 6 h; indeed, a remarkably dilated RER, numerous swollen mitochondria, and a relevant loss of secretory granules were observed in the cytoplasm of beta cells. Several large residual bodies and autophagic vacuoles containing no clearly identifiable structures (most likely degraded membranes) were also detected. The accumulation of autophagic vacuoles and residual bodies induced an engulfment in some beta cells, that however was not associated to clear signs of cell death such as loss of plasma membrane integrity (Fig. 9C).

When human islets were exposed for 6 h to a combination of 0.5 mM PA and 25 mM glucose, the ultrastructural beta-cell alterations were generally not very different from those induced by PA alone (Fig. 8D). After 24 h of combined exposure to 0.5 mM PA and 25 mM glucose, the ultrastructure of beta cells was more strongly altered. Nuclei were frequently distorted with periferically condensed chromatin, suggesting a pre-apoptotic situation. The RER was very dilated and its membranes were distributed mainly around the nuclei but also throughout the cytoplasm. The mitochondria were definitely swollen, round in shape, with a dispersed matrix and fragmented cristae. Few and small mature beta granules were observed. Several large autophagic vacuoles containing identifiable organules, such as mitochondria and lipid-like droplets, could be also observed, together with numerous and large residual bodies (Fig. 9D).

Quantitative analysis by morphometric methods confirmed that increased autophagic vacuoles (AV) formation and remarkable dilation of RER occurred after 24 h incubation with 0.5 mM PA, but not with 25 mM glucose alone. Indeed, the volume density of AV and RER in beta cells of human islets was the following (expressed as ml%): **AV**: C 0.3±0.20; 0.5 mM PA 1.1±0.25 (p<0.05 vs C); 0.25 mM glucose 0.1±0.10; PA+Glucose 1.0±0.34 (p<0.05 vs C); **RER**: C 0.4±0.12; 0.5 mM PA 4.4±0.70 (p<0.05 vs C); 25 mM Glucose 0.5±0.17; PA+Gluc 3.5±0.55 (p<0.05 vs C).

**Figure 10:**
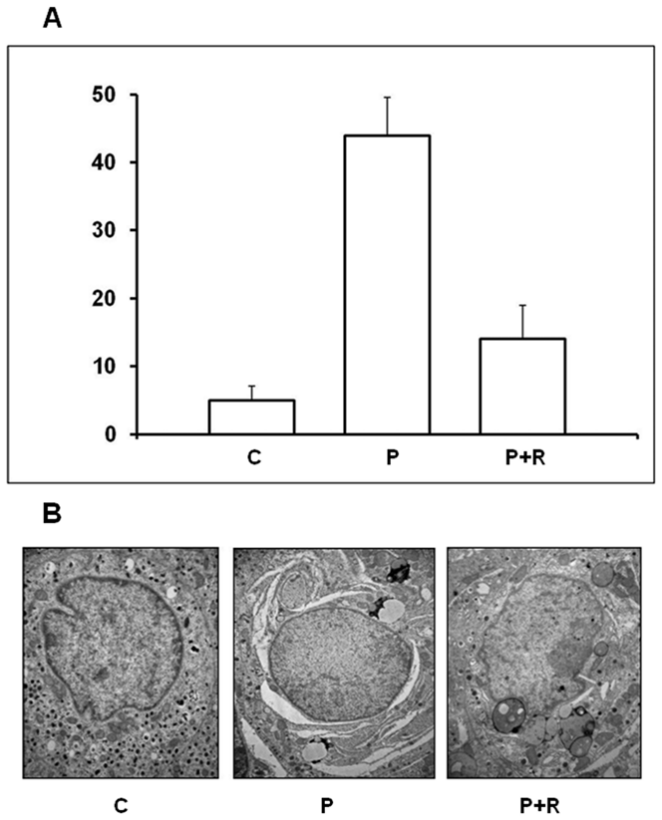
**A**) Ultrastructural morphometric analysis of beta-cell death in human islets after 48 h exposure to 0.5 mM palmitic acid ±10 ng/ml rapamycin. C = control; P = 0.5 mM palmitic acid; P+R = 0.5 mM palmitic acid plus 10 ng/ml rapamycin. Mean of 3–4 samples ± SEM. Statistical analysis (Bonferroni's test): * p<0.05 vs control and vs palmitate+rapamycin; # p>0.05 vs palmitate. **B**) Electron microscopy of isolated human islets in control conditions (left) and after 48 h incubation with 0.5 mM PA (center) or 0.5 mM PA+10 ng/ml rapamycin (right). (magnification: 10.000×).

Finally, we wanted to better ascertain whether a prolonged exposure to PA would determine a clear-cut increase of beta cell death in human islets. To this purpose, we morphometrically analysed samples of isolated human islets processed for electron microscopy after 48 h exposure to 1.0 mM PA following the morphological criteria mentioned above in the “Material and Methods” section. We found that the percent of beta cells undergoing cell death was significantly increased with respect to controls (Fig. 10). Interestingly, when human islets were exposed for 48 h to 0.5 mM PA in the presence of 10 ng/ml rapamycin, a well known pharmacological activator of autophagy, the percent of dead cells was significantly reduced (Fig. 10A). Ultrastructurally, in the beta cells still alive after 48 h exposition to PA, an impressive dilation of the ER was constantly observed, associated with the presence of several quite enlarged autophagic vacuoles and dense bodies. The beta granules were drastically reduced and swollen mitochondria could be observed (Fig. 10B, central panel). Interestingly, when rapamycin was present, the main difference with respect to PA alone was represented by a significant reduction of the extension and dilation of RER. Several dense bodies were still detectable, whereas autophagic vacuoles were evidently reduced in number (Fig. 10B, right panel), probably as a result of a rapamycin-induced increased rate of autophagic fluxes. These observations, were validated through morphometric evaluation of the volume density of autophagic vacuoles (AV), and RER in beta cells of human islets in the above mentioned experimental conditions, i.e. after 48 h incubation with 0.5 mM PA, in the presence or absence of 10 ng/ml rapamycin (**AV**: C 0.1±0.09; 0.5 mM PA 1.2±0.14 (p<0.05 vs C); PA+rapamycin 0.1±0.06 (p<0.05 vs PA); **RER**: C 0.8±0.23; 0.5 mM PA 2.5±0.48 (p<0.05 vs C); PA+rapamycin 0.7±0.25 (p<0.05 vs PA); volume density is expressed as ml%).

## Discussion

In this research we have addressed the question of whether increasing concentrations of glucose and/or palmitic acid could activate the process of autophagy in pancreatic beta cells in three different experimental models (i.e. INS-1E cells and pancreatic rat and human isolated islets). We have decided to explore these two stimuli in order to mimic *in vitro* the two major metabolic alterations typically observed in type 2 diabetes. In particular, we chose palmitate as free fatty acid because it has been demonstrated that palmitate but not oleate induces significant ER stress contributing to beta-cell apoptosis [41]. Our results clearly indicate that palmitate concentrations as high as 0.5 or 1.0 mM are able to markedly activate autophagy in beta cells (as indicated by several morphological and biochemical markers), whereas high glucose alone is ineffective.

Autophagy is a degradative mechanism that can be induced by starvation or other form of nutrient deprivation to supply substrates for cellular energy generation [5]. Autophagy also serves as a catabolic pathway to recycle excessive or damaged intracellular organelle such as mitochondria [6], [7]. Thus, autophagy can act as a housekeeping mechanism in the absence of stress, while in stress conditions it exerts a crucial protective role [42]. For a long time, autophagy has been correlated almost exclusively to protein and organelle turnover, by which amino acids could be made available from endogenous sources during nutrient starvation. However, evidences have recently emerged linking autophagic function to other types of metabolic stress, including that determined by carbohydrates and lipids derangements [42]. These novel findings support the idea that autophagy plays a very important role in the regulation of cellular metabolic capabilities [43]–[45]. With regard to lipid metabolism, recent studies have shown that lysosomal lipases contribute to intracellular lipolysis through the degradation of lipid droplets delivered to lysosomes not only through an endocytotic pathway but also through macroautophagy in a process termed lipophagy [46]. Intracellular lipids were not previously considered autophagic substrates, but the striking similarities between lipolysis and autophagy (that are both activated under nutrient deprivation), together with the existence of lysosomal lipases, suggested a possible link between the two pathways [47]. It has also been shown that defective autophagy in mice leads to increased concentrations of hepatic tryglicerides and cholesterol, presence of larger and more abundant intracellular lipid droplets, and liver enlargement [46]. Disruption of autophagy leads to intracellular lipid accumulation also in endothelial cells, lymphoblasts, dendritic cells, glial cells and even in neurons [48]. Altogether, these results suggest a generalized function of autophagy in cellular lipid handling. This function may result particularly advantageous during nutrient deprivation, where its activation persists well beyond the 6–8 h limit established for macroautophagy-dependent proteolysis [46]. Furthermore, besides its activation during energetically demanding conditions, autophagy could be also used by various cell types to handle massive affluence of lipids [45]. Our results, showing a marked activation of autophagy in pancreatic beta cells exposed to elevated concentrations of free fatty acids such as palmitate, in agreement with previous results [23], can clearly be well integrated in this scenario. Interestingly, in our experimental conditions, high glucose concentrations were per se unable to activate autophagy and did not substantially enhance the effect of FFA when used in combination.

The observed differential effect of glucose and PA on autophagy activation in beta cells raises the question of the mechanism responsible for the PA-induced alterations. It has been widely demonstrated that free fatty acids are able to trigger ER stress in beta cells via an NF-kB and nitric oxide-independent mechanism [49], probably through changes in ER Ca^++^ handling [50]. On the other hand, both ER stress and a rise in the free cytosolic calcium have been shown to be potent inducers of macroautophagy [51], [52]. In agreement with these observations, our results clearly indicate that the exposition of beta cells to PA, but not to high glucose, is associated with the ultrastructural evidence of a remarkable dilation of the ER. The differential effect of PA and glucose on ER dilation is also quantitatively supported by the morphometric measurement performed in the exposed human islets. At this regard, it is also interesting to mention our previous report that PA and high glucose can differentially regulate the expression of several molecular markers associated with ER stress in isolated human islets [27], [53]. Therefore, the activation of an ER stress response represents a likely candidate for the mechanism of the PA-induced autophagy activation. However, the available data are not sufficient to exclude alternative hypotheses, such as the involvement of G-protein coupled receptors that have been recently reported to play a role in the PA-induced beta-cell dysfunction [54].

Among the metabolic factors that have been associated with an increased prevalence of several diseases, including type 2 diabetes, free fatty acids have attracted much attention, especially in the context of lipid-induced pancreatic beta cell dysfunction (lipotoxicity) [55]. Indeed, it has been demonstrated that high levels of FFA may both impair insulin secretion [56], [57] and induce cell death by apoptosis [35], [58], [27]. Thus, the observation that exposure to high palmitate is associated with a significant and long-lasting activation of autophagy, rises the question of the role played by autophagy in diabetes-associated beta cell dysfunction and death. In this regard, autophagy, like many other cellular defence mechanisms, can be considered as a double-edged sword.

On one side, it has been recently demonstrated that constitutive autophagy plays important roles in the maintenance of pancreatic beta-cell homeostasis. In particular, specific deletion of Agt7 in pancreatic beta cells has been shown to be associated with increased apoptosis and decreased proliferation of beta cells, leading to a decrease in beta-cell mass [21], [22]. Functional analysis further revealed that both basal and glucose-stimulated insulin secretion were defective in primary islets of Atg7-defective mice [21], [22], [59]. Furthermore, autophagy has been reported to control insulin content and ubiquitin aggregate formation in response to oxidative stress or hyperglycemia in beta cells [16], [60]. The beneficial role of autophagy in cells such as pancreatic beta cells, in which vigorous and sustained protein synthesis and secretion occur, is probably attributable to its protective role against ER stress [61], [62]. In this regard, it is important to mention that over the past years, ER stress response has been identified as a molecular mechanism of lipotoxicity that may play a role in human diabetes [63], [64]. Saturated and, to a lesser extent, unsaturated FFAs have been shown to trigger ER stress in beta cells through changes in ER Ca^2+^ handling [65], [66] and/or through activation of the PKR-JNK1 pathway [67]. In this scenario, it seems of particular interest the observation of the present study showing a remarkably dilated endoplasmic reticulum in beta cells exposed to high palmitate but not to high glucose. Thus, increased autophagy in response to fatty acids might be considered as a protective mechanism from lipotoxicity. Our results, showing the immunohistochemical co-localization of an autophagic vacuole marker (LC3) with a lysosomal marker (catepsin D) in INS-1 cells after 6 h of palmitate treatment indicate that autophagic fluxes seem to be indeed not blocked by palmitate in beta cells. This conclusion is further supported by the subsequent attenuation of LC3 immunohistochemical signal after 24 h of palmitate treatment, suggesting its accomplished lysosomal degradation. The question regarding the effect of fatty acids (and in particular palmitate) on autophagic fluxes in pancreatic beta cells is still somehow controversial. Indeed, whereas Las et al. [55] recently reported that palmitate suppress autophagic fluxes in association with a decrease in lysosomal activity, Komiya et al. [67] on the contrary concluded from their results that palmitate activates autophagic fluxes in most cells. However, our results, while suggesting that autophagosome-lysosome fusion is probably not affected, do not rule out the possibility that subsequent complete degradation might be impaired.

On the other hand, at the level of both the cell and the organism, autophagy can paradoxically have either pro-survival or pro-death functions depending on the context [68]. At the cell level, several recent studies indicates that autophagy itself may be a mechanism of caspase- and apoptosis-independent cell death [11]. Overall, evidence indicates that autophagy is a cell survival pathway that can also mediate cell death under certain conditions, e.g. when autophagy is overactivated [69]. At the level of the organism, altered autophagy may be implicated in several diseases, such as cancer, neurodegenerative disorders, autoimmune conditions like Crohn's diseases and rheumatoid arthritis, heart disease and infection [13], [31]. Recently, a role for alterations of autophagy in the pathogenesis of pancreatic diseases, including diabetes, has been proposed [70], [71]. At this regard, our previous findings concerning the observation of massive vacuole overload in beta cells of type 2 diabetic patients [24] clearly suggest that altered autophagy may contribute to the loss of beta-cell mass in diabetes.

Whereas the question of whether autophagy could play a role in FFA-induced cell death remains to be fully elucidated, our preliminary results indicate that rapamycin, a potentiator of autophagy, seems to have a pro-survival action against PA-induced beta-cell apotosis. This would therefore favours the view that in given situations promoting autophagy could be beneficial. In agreement, Choi et al. [23] recently reported that the augmentation of the autophagic system, obtained either by rapamycin or an inhibitor of Akt, had a protective effect on palmitate-induced INS-1 cell death. However, these Authors also showed that exposure of palmitate-treated INS-1 cells to 3-methyladenine, a well known inhibitor of autophagy, resulted slightly beneficial. These apparently contradictory results deserve to be interpreted cautiously. Indeed, 3-MA, besides its well known ability to block autophagy by inhibiting autophagosome formation, has been also reported to exert direct anti-apoptotic effects by unknown mechanisms [72]. Further caution is suggested by our data regarding the effect of rapamycin on ER dilation in beta cells of human islets exposed to PA for 48 h. The drastic reduction of ER dilation might indeed suggest that rapamycin could influence cell survival not only by modulating autophagy but also indirectly through a decrease of ER stress, in agreement with very recently reported results [73].

In conclusion, the present study described several features regarding the effects of exposure to glucose and/or palmitate on beta cell autophagic processes, showing that different nutrients affects autophagy differently in our conditions. Our data also propose that fostering autophagy may improve the defence of beta cells against metabolic insults.

Our results clearly indicate that in pancreatic beta cells increased palmitate levels could represent a powerful activator of the autophagic process, whereas high glucose levels are ineffective at this regard. These findings have been obtained using different experimental techniques (MDC fluorescence, immunoblotting, immunofluorescence, electron microscopy), in accordance with the internationally acknowledged guidelines for the study of autophagy [74], and have been confirmed in both an insulin-secreting cell line (INS-1E) and in isolated rat and human pancreatic islets. The molecular mechanism of palmitate-induced activation of autophagy remains to be fully elucidated, but our electron microscopy observations, together with similar results reported elsewhere [65], strongly indicate that ER stress might play a crucial pathogenic role. More work remains to be done in order to fully appreciate the significance of autophagy in the mechanisms underlying beta-cell damage in diabetes (in other words, whether autophagy must be regarded as a cell survival mechanism or an inducer of non-apoptotic cell death), but the present results appear helpful for our understanding of the metabolic conditions that can activate autophagy in type 2 diabetes.
