# Correction: Palmitate Activates Autophagy in INS-1E β-Cells and in Isolated Rat and Human Pancreatic Islets

**DOI:** 10.1371/journal.pone.0122235

**Published:** 2015-03-16

**Authors:** The PLOS ONE Staff

Fig. 2A incorrectly appears as a duplicate of Fig. 1A. The authors have provided a corrected version of Fig. 2 here.

**Fig 2:**
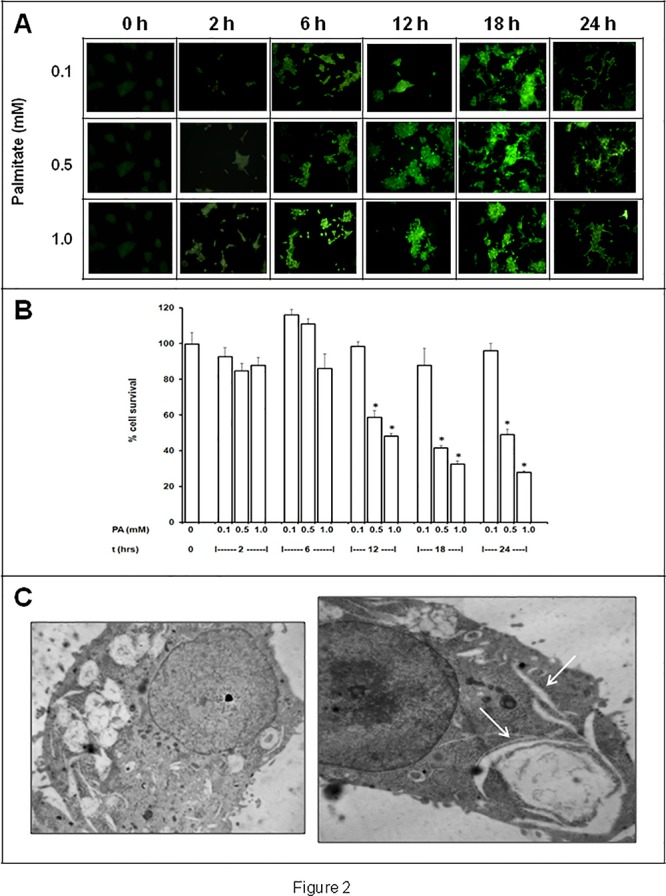
Effects of various incubation times (2–24 h) different palmitic acid (PA) concentrations (0.1, 0.5 and 1.0 mM) in INS-1E cells. A) MDC fluorescence, as indicator of autophagosome formation; B) cell viability; C) ultrastructure (0.5 mM PA for 24 h) (magnification: 10.000×).
